# Photothermally Responsive Poly(vinyl alcohol)/Polyaniline Nanoparticle Composite Hydrogels Prepared by a Facile Aqueous Route

**DOI:** 10.3390/polym18131638

**Published:** 2026-07-01

**Authors:** Ernesto S. Battaglia, Eduart Gutiérrez-Pineda, César A. Barbero, Gustavo A. Abraham, Sergio E. Moya, Silvestre Bongiovanni Abel

**Affiliations:** 1 Research Institute for Materials Science and Technology (INTEMA), National University of Mar del Plata (UNMdP)-National Scientific and Technical Research Council (CONICET), Av. Colón 10850, Mar del Plata 7600, Argentina; 2 Center for Cooperative Research in Biomaterials (CIC biomaGUNE), Basque Research and Technology Alliance (BRTA), Paseo Miramon 182, 20014 Donostia San Sebastián, Spain; 3 Research Institute for Energy Technologies and Advanced Materials (IITEMA), National University of Rio Cuarto (UNRC)-National Scientific and Technical Research Council (CONICET), Ruta Nacional N° 36 Km 601, Río Cuarto 5800, Argentina; 4 Department of Chemical and Food Engineering, Faculty of Engineering, National University of Mar del Plata (UNMdP), Av. Juan B. Justo 4302, Mar del Plata 7600, Argentina

**Keywords:** nanocomposite hydrogels, poly(vinyl alcohol), polyaniline nanoparticles, photothermally active platforms

## Abstract

Here, we report a facile, reproducible, fully aqueous route to fabricate citric acid–crosslinked poly(vinyl alcohol) (PVA) composite hydrogels incorporating polyaniline nanoparticles (PANI-NP) of ca. 200 nm mean diameter and polydispersity index (PDI) below 0.2, synthesized directly in water. Nanocomposites incorporating 2, 3, and 5% *w*/*w* PANI-NP were thoroughly characterized in terms of thickness (obtaining materials of approximately 500 µm), morphology, spectroscopic and thermal properties, surface properties, swelling behavior, and nanomechanical behavior assessed by atomic force microscopy (AFM) operating in Peak Force Quantitative Nanomechanical Mapping (PF-QNM) mode. Incorporation of PANI-NP progressively increased the elastic modulus of the composites (from 794 MPa for neat PVA to values exceeding several GPa at 3–5% *w*/*w* loading) and modified swelling capacity to values as low as 140% (from 247% for neat PVA), reflecting nanoscale interfacial interactions. Notably, the hydrogel composites exhibited significant photothermal activity under low-power near-infrared (NIR) LED irradiation (850 nm, 90 mW cm^−2^), achieving temperature increases of up to 13.7 °C even at low PANI-NP loadings, with a stable and reproducible response across multiple heating–cooling cycles. Overall, this work establishes a straightforward, water-based fabrication platform for structurally stable, photothermally active nanocomposites with promising potential in light-responsive smart material applications.

## 1. Introduction

Polymer networks provide a versatile framework for designing functional materials, they allow for the incorporation of nanoparticles, fillers, or molecular components to tailor their physical, mechanical, and thermal properties [1]. Among the different types of polymer networks, hydrogels constitute a particularly relevant subclass, defined as three-dimensional hydrophilic networks capable of absorbing and retaining large amounts of water while maintaining their structural integrity due to the presence of crosslinks [2,3]. Owing to their high water content, soft structure, and tunable physicochemical properties, hydrogels have attracted considerable attention in areas ranging from biomedical applications to sensors and food packaging [1,4,5,6].

In recent decades, poly(vinyl alcohol) (PVA) hydrogels have demonstrated outstanding properties which make them versatile polymeric materials. Among others, their good biocompatibility, tunable mechanical properties, optical transparency, biodegradability, and high-water retention capacity are remarkable features that have positioned PVA as a material of choice for applications in biomedicine, sensors, wound dressings, and functional coatings [7,8]. Nevertheless, several crosslinking methods reported in the literature present notable drawbacks, including reliance on organic solvents, toxic crosslinker agents, and environmentally unfavorable synthetic routes, which constrain their broader applicability [9,10]. In this regard, citric acid (CA) has emerged as a promising green crosslinking agent, owing to its low toxicity, low cost, and capacity to form ester bonds with the hydroxyl groups of PVA chains, providing structural stability to the polymer network while preserving its biocompatibility [11,12,13].

The incorporation of nanoparticles into hydrogel matrices has become a powerful and widely adopted strategy to overcome the intrinsic limitations of neat polymer networks and expand their functional scope. Depending on the nature of the nanofiller, nanocomposite hydrogels can exhibit enhanced mechanical properties, improved thermal stability, and tailored responsiveness to external stimuli such as light, pH, temperature, or electric fields [14,15]. Beyond property enhancement, functional nanoparticles enable the design of application-specific platforms by imparting functionalities absent in the pristine hydrogel matrix, including electrical conductivity, optical activity, antimicrobial behavior, or photothermal response [16,17,18,19]. To this end, a wide variety of nanofillers has been explored, including inorganic nanoparticles such as gold, silver, and iron oxide, carbon-based materials such as graphene and carbon nanotubes, and organic conducting nanoparticles based on electroactive polymers, among others [20,21,22,23]. In all cases, the degree to which these nanofillers effectively interact with the polymer network at the nanoscale remains a key factor governing the ultimate material performance.

Within this context, conducting polymer-based nanofillers offer a particularly attractive alternative due to their intrinsic electroactivity and tunable physicochemical properties. Among the different electroactive nanofillers, polyaniline (PANI) has attracted considerable attention due to its unique combination of properties [24]. PANI can exist in different oxidation and protonation forms, including the fully reduced leucoemeraldine, the half-oxidized emeraldine base and its protonated form (emeraldine salt), and the fully oxidized pernigraniline. Of these, the emeraldine salt state (PANI-ES) is the most chemically stable form and therefore it is the most relevant for practical applications. Interconversion among these states is reversible and responds to both redox and pH stimuli, conferring PANI with electrochromic and stimuli-responsive behavior [25,26,27]. Furthermore, PANI-ES exhibits a broad absorption band in the near-infrared (NIR) region associated with polaron transitions, which enables efficient conversion of NIR radiation into heat, making it a promising organic photothermal agent. These properties are preserved when PANI is processed in nanoparticulate form (PANI-NP), which additionally offers advantages in terms of colloidal stability, aqueous dispersibility, and compatibility with polymeric matrices [28]. In this context, PANI-NP have been previously synthesized, characterized, and evaluated as photothermal agents and electroactive nanofillers by our group, providing a well-defined nanomaterial platform for incorporation into functional hydrogel composites [18,24].

Photothermal materials capable of converting NIR light into heat have gained increasing interest as promising functional platforms for light-responsive systems [29,30,31]. Although some approaches combining electroactive nanoparticles with polymeric matrices have been reported, most studies on conductive PVA/PANI composites have employed PANI polymerized in situ within the matrix or in fiber form rather than as discrete preformed nanoparticles. In the few cases where PANI-NP have been incorporated into hydrogel matrices, alternative polymer matrices or crosslinking strategies relying on e-beam irradiation or toxic reagents such as glutaraldehyde have been employed [32,33]. Moreover, the photothermal performance under low-power NIR irradiation and the nanoscale mechanical characterization via AFM/PF-QNM of PVA/PANI-NP composite hydrogels have been largely unexplored, even in previous work from our group focusing on related systems [18]. In this context, herein we report a straightforward and reproducible aqueous method to fabricate citric acid-crosslinked PVA composite hydrogels incorporating PANI-NP as electroactive and photothermal nanofillers. The developed composites were systematically characterized in terms of morphology, spectroscopic, thermal, and nanomechanical properties, surface behavior, and swelling capacity. Furthermore, their photothermal performance under low-power NIR LED irradiation was evaluated, demonstrating their potential as structurally robust, stable, and photothermally active platforms for light-responsive systems and smart material applications.

## 2. Materials and Methods

### 2.1. Chemicals

Poly(vinyl alcohol) (PVA Mw: 31,000–50,000, 99% hydrolyzed) was purchased from Sigma Aldrich (St. Louis, MO, USA) as a solid and dissolved in deionized water prior to use. Citric acid was provided by Anedra Research S.A. (Buenos Aires, Argentina). Aniline hydrochloride (ANI-HCl), used as monomer for PANI-NP synthesis, was acquired from Sigma Aldrich. Poly(vinyl pyrrolidone) (PVP Mw: 360,000), used as a nanoparticle stabilizer, and ammonium persulfate (APS) were also purchased by Sigma Aldrich and Biopack (Buenos Aires, Argentina), respectively. Glycerol, incorporated to increase viscosity of PVA solution, was supplied by BioBasic (Markham, ON, Canada). All reagents were used as received without further purification. Milli-Q water was used throughout all experiments.

### 2.2. Nanocomposite Hydrogels Synthesis

**Scheme 1:**
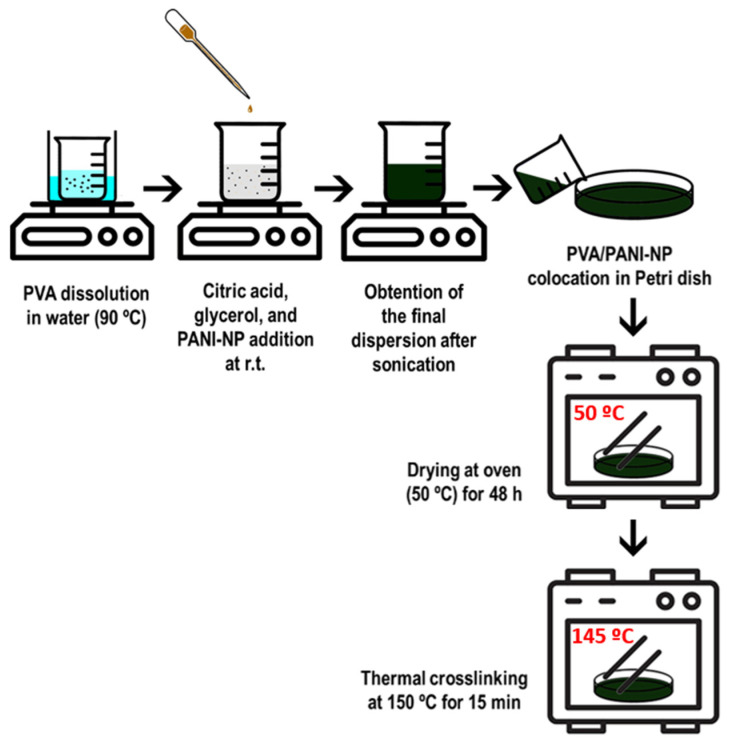
Representative scheme of the general procedure for the fabrication of PVA/PANI-NP composite hydrogels.

PVA/PANI-NP composite hydrogels were fabricated following the procedure outlined below. PANI-NP were first synthesized as previously reported [24]. Briefly, ANI-HCl was dissolved in water at a concentration 0.2 mol L^−1^ and then mixed with a 2% *w*/*v* aqueous PVP solution, followed by addition of the polymerization initiator APS (10 mL 0.25 mol L^−1^). The resulting PANI NP were purified by dialysis for 48 h and subsequently freeze dried prior to use. The dried PANI-NP were then added to the PVA solution (8 mL, 18% *w*/*v*) containing citric acid (20% *w*/*w*) at three different loadings: 2, 3 and 5% *w*/*w*. The resulting dispersions were gently stirred at 300 rpm for 24 h at room temperature, with three sonication cycles (15 min each) applied during the first hour of mixing to ensure homogeneous nanofiller distribution. Each dispersion was then cast into Petri dishes, degassed to remove air bubbles, and dried in an oven at 50 °C for 48 h. The dried films were subsequently crosslinked by thermal treatment at 145 °C for 15 min, yielding the final PVA/PANI-NP composite hydrogels. Table 1 summarizes the composition of each sample, and a schematic representation of the full fabrication procedure is presented in Scheme 1.

### 2.3. Materials Characterization

#### 2.3.1. Morphology and Thickness

Prior to incorporation into the composite hydrogels, PANI-NP were characterized morphologically by electron microscopy. Scanning electron microscopy (SEM) micrographs were obtained using an FEI-Quanta 200 scanning electron microscope (FEI, Hillsboro, OR, USA) operated at an accelerating voltage of 5 kV. Samples were sputter-coated with a thin chromium layer prior to imaging to prevent charge accumulation. Particle diameters were determined from SEM images using ImageJ Pro^®^ software (version 1.54). Additionally, transmission electron microscopy (TEM) analysis was performed using a JEOL JEM 2100 instrument (JEOL, Tokyo, Japan) operated at 200 kV and equipped with a LaB_6_ filament. TEM samples were prepared by depositing a controlled volume of the PANI-NP dispersion onto copper grids and allowing solvent evaporation under ambient conditions. The thickness of the neat PVA and PVA/PANI-NP composite hydrogel films was measured using an AOS Induction Sensor Digital Electronic Caliper (Mitutoyo, Kanagawa, Japan). For each sample, measurements were taken at eight different locations (*n* = 8), and the mean thickness was reported.

#### 2.3.2. Dynamic Light Scattering (DLS)

The colloidal stability and size distribution of PANI-NP dispersions was assessed by DLS using a Malvern Zetasizer Nano-S90 instrument (Malvern, Malvern, UK), operating at a detection angle of 90° and room temperature. Measurements were performed over a pH range of 4–10, with the pH adjusted by addition of 0.1 mol L^−1^ NaOH or 0.1 mol L^−1^ HCl prior to dilution in Milli-Q water. For each condition, three independent measurements of 30 runs each (n = 3) were conducted to determine the mean hydrodynamic diameter and polydispersity index (PDI).

#### 2.3.3. UV–Visible Spectroscopy

UV–Visible absorption spectra of PANI-NP dispersions at varying pH values were recorded using an Agilent 8453 spectrophotometer (Agilent, Santa Clara, CA, USA) over a wavelength range of 300–1100 nm. Quartz cuvettes with a 1 cm optical path length were used, and Milli-Q water served as the blank reference.

#### 2.3.4. Raman Spectroscopy

Raman spectra of neat PVA and nanocomposite hydrogels were acquired using an InVia Reflex confocal Raman microscope (Renishaw, Wotton-under-Edge, UK), coupled to a Leica optical microscope (Leica, Wetzlar, Germany) with an XYZ motorized stage (Prior, Cambridge, UK) and equipped with a Peltier-cooled, front-illuminated 1024 × 512 CCD detector and a 1200 lines mm^−1^ diffraction grating. All measurements were performed directly on the film surface under ambient conditions using a 785 nm laser excitation source operated at 1% laser power and a 10× objective. Spectra were collected from multiple regions of each film to assess spectral reproducibility and local heterogeneity: each reported spectrum represents the average of at least three measurements acquired at different locations on the same sample.

#### 2.3.5. Topography, Roughness and Nanomechanical Properties

Surface topography, roughness, and nanomechanical properties were characterized using a Bruker Multimode 8 HR atomic force microscope (Bruker, Billerica, MA, USA) equipped with a Nanoscope V controller (Bruker, Billerica, MA, USA), operated in PeakForce Quantitative Nanomechanical Mapping (PF-QNM) mode. Measurements were carried out in air at room temperature (25 °C) on samples sectioned into 5 mm × 5 mm pieces and mounted on glass substrates. Images were acquired over 2.5 × 2.5 µm^2^ areas at a resolution of 512 × 512 pixels, a scan rate of 0.5 Hz, and a PeakForce modulation frequency of 2 kHz, using a Bruker Nanoscope software (Nanoscope 2.0).

Nanomechanical mapping was performed using RTESPA-525 probes (Bruker, Billerica, MA, USA) following the calibration protocol previously described in PF-QNM studies [34,35,36]. Specifically, the photodetector deflection sensitivity was calibrated against a sapphire standard, the cantilever spring constant (k) was determined by the thermal noise method, and the tip radius (R) was verified using a polycrystalline titanium roughness standard. PeakForce setpoints were adjusted to maintain indentation depths of approximately 5 nm, below 10% of the total film thickness, to minimize substrate contributions to the measured response. Apparent elastic modulus maps were obtained by the fitting force–distance curves with the Derjaguin–Muller–Toporov (DMT) contact mechanics model, as implemented in the Bruker PF-QNM analysis workflow [34]. Adhesion maps were recorded simultaneously.

#### 2.3.6. Water Contact Angle (WCA)

The surface hydrophilicity of the neat PVA and PVA/PANI-NP composite hydrogels was evaluated by static water contact angle (WCA) measurements using a ramé-hart goniometer (Succasunna, NJ, USA). A water drop of 5 µL was deposited onto the hydrogel surface and the contact angle was recorded over 180 s. Measurements were performed in triplicate for each sample, and image analysis was carried out using ImageJ Pro^®^ software.

#### 2.3.7. Swelling Behavior

The equilibrium swelling capacity of the hydrogels was evaluated gravimetrically. Dried samples were accurately weighed in an analytical balance and subsequently immersed in water at room temperature. After 12 h, upon reaching equilibrium swelling, samples were removed, gently blotted with filter paper to remove surface-adherent water, and reweighed. The equilibrium swelling degree (%) was calculated according to Equation (1).

(1) $$\mathrm{Equilibrium}\;\mathrm{Swelling}\;(\%)=\frac{W_{\mathrm{eq}}-W_{0}}{W_{0}}\times 100$$

where $W_{\mathrm{eq}}$ is the weight of the hydrogel at equilibrium swelling and $W_{0}$ is the initial dry weight. All measurements were performed in quintuplicate (*n* = 5).

#### 2.3.8. Thermogravimetric Analysis (TGA)

The thermal stability of neat PVA hydrogel and PVA/PANI-NP nanocomposite samples in the dry state were assessed by thermogravimetric analysis using a Shimadzu TGA-50 instrument (Shimadzu, Kyoto, Japan). Approximately 10 mg of each sample was heated from room temperature to 700 °C under a nitrogen atmosphere at a heating rate of 10 °C min^−1^.

### 2.4. Evaluation of Photothermal Performance

The photothermal response of PVA/PANI-NP composite hydrogels was evaluated by irradiating circular samples (diameter = 0.5 cm), placed in a 96-well plate, with an 850 nm LED at a power density of 90 mW cm^−2^. A water layer was interposed between the LED source and the wells to filter direct thermal radiation and ensure that the recorded temperature rise was attributable solely to the photothermal activity of the composites. Sample surface temperature was monitored using an infrared thermal imaging camera (Testo 868, Testo SE & Co. KGaA, Titisee-Neustadt, Germany) at 2 min intervals over a total irradiation period of 20 min. Neat PVA hydrogel was included as a negative control to confirm that the observed photothermal effect originates from the PANI-NP component. The stability and reproducibility of the photothermal response were further assessed by subjecting each composite formulation to sequential heating–cooling cycles under the same irradiation conditions.

## 3. Results

**Figure 1:**
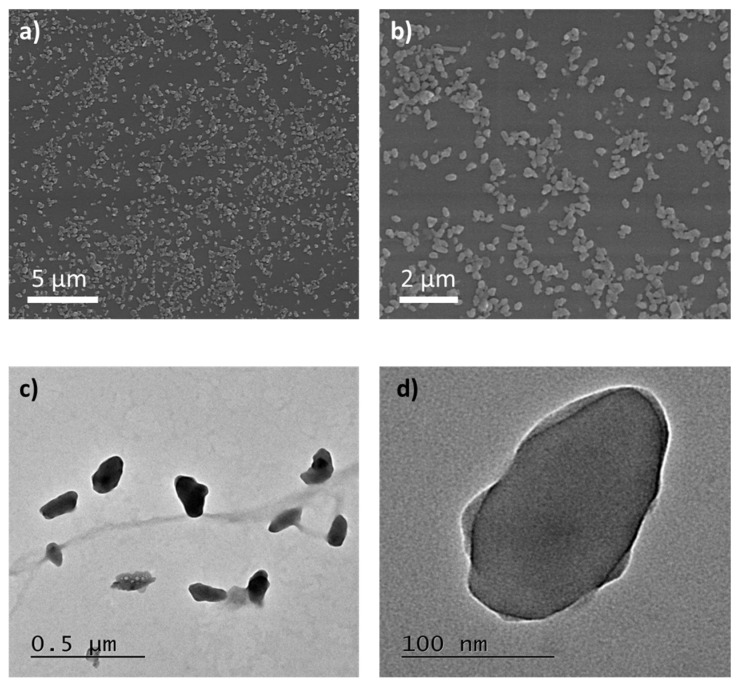
(**a**,**b**) SEM images of PANI-NP. Scale bar: (**a**) 5 µm, (**b**) 2 µm; (**c**,**d**) TEM images of PANI-NP. Scale bar: (**c**) 0.5 µm, (**d**) 100 nm.

PANI-NP were characterized by electronic microscopy to confirm their successful formation of nanoparticles and assess their morphological features. SEM micrographs (Figure 1a,b) reveal a relatively homogeneous particle distribution, with a slight degree of agglomeration also observed. The nanoparticles display a quasi-spherical to irregular shape, consistent with PANI-NP synthesized under analogous conditions. In addition, TEM images (Figure 1c,d) corroborate these findings, revealing particles in the nanometric scale with slightly elongated and non-regular spherical shape. The observed morphology and moderate polydispersity are not expected to adversely affect the subsequent incorporation of PANI-NP into the PVA matrix or the structural integrity of the resulting composite hydrogels.

**Figure 2:**
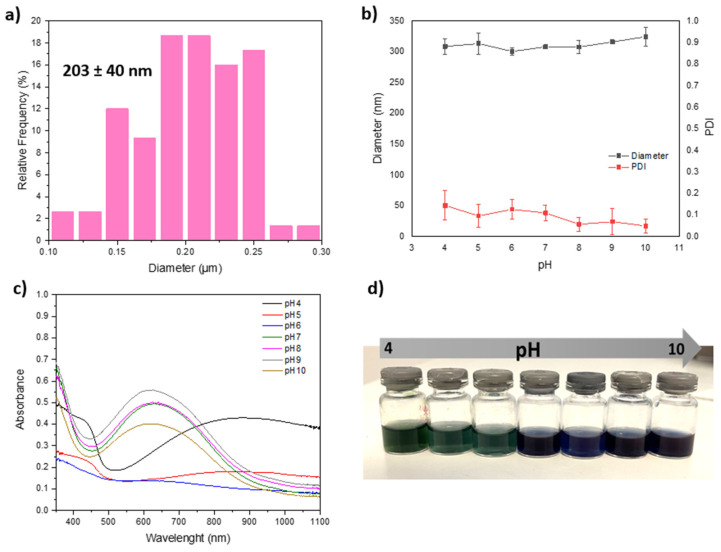
(**a**) Size distribution of PANI-NP determined from SEM images; (**b**) average diameter and polydispersity index (PDI) measured at different pH values; (**c**) UV–Visible spectra for PANI-NP at different pH values; (**d**) photograph showing the PANI-NP dispersion at the evaluated pH values.

Particle size analysis from SEM images yielded a size distribution (Figure 2a) with a mean particle diameter of 203 ± 40 nm. The hydrodynamic diameter and polydispersity index (PDI) of PANI-NP were further evaluated by DLS as a function of pH over a range of 4–10 (Figure 2b). No significant variation in the hydrodynamic diameter was observed across this pH range, indicating the protonation/deprotonation equilibria of the electroactive polymer backbone do not appreciably affect interparticle interactions in aqueous dispersion. As expected, the hydrodynamic diameter exceeded the value obtained from SEM, consistent with the contribution of the hydration shell and the difference between dry and solution-phase measurements. In all cases, PDI values remained below 0.2 confirming a narrow and a homogeneous particle size distribution. Given their proposed application as photothermal agents, the optical properties of PANI-NP were assessed by UV–Visible spectroscopy as a function of pH (Figure 2c) [24]. The spectra display the characteristic absorption features of PANI: a π–π* transition band in the 300–350 nm region and a broad polaron absorption band extending into the NIR region. The latter is most intense under acidic conditions and progressively diminishes with increasing pH, accompanied by a slight hypsochromic shift. This behavior is consistent with the electronic transition from the protonated PANI (emeraldine salt) to the deprotonated emeraldine base form, in agreement with previously reported results, confirming the capacity of these electroactive nanoparticles for efficient NIR absorption under acidic and near-neutral pH conditions [28]. In addition, FTIR analysis of PANI-NP (Figure S1) confirms the presence of the characteristic absorption bands of PANI-ES, including C=C stretching of quinoid (1560 cm^−1^) and benzenoid (1480 cm^−1^) rings, C-N stretching (region of 1300 cm^−1^), and the characteristic polaron band at approximately 1140 cm^−1^. Figure 2d shows representative photographs of the PANI-NP dispersions corresponding to the samples analyzed by UV–Visible spectroscopy and DLS, illustrating the visible color changes associated with the pH-dependent oxidation state transitions. Finally, TGA analysis (Figure S2) confirms the thermal stability of PANI-NP up to temperatures close to 200 °C, indicating that the nanofiller would not be affected during the crosslinking process employed for hydrogel fabrication.

**Figure 3:**
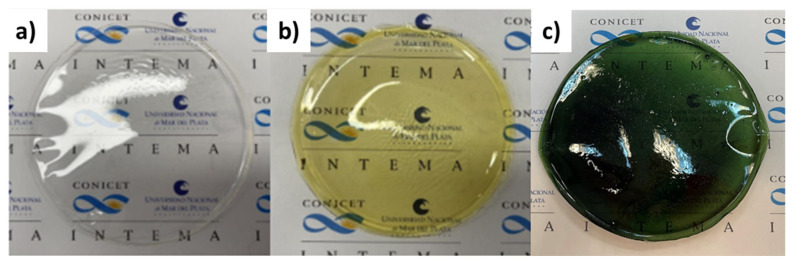
Photographs of different stages of the materials: (**a**) PVA hydrogel before crosslinking; (**b**) PVA hydrogel after thermal crosslinking; (**c**) PVA/PANI NP composite hydrogel.

Building on the physicochemical characterization of PANI-NPs described above, PVA/PANI-NP composite hydrogels were fabricated at three nanoparticle loadings (2, 3, and 5% *w*/*w*) following the procedure outlined in Section 2.1. Representative photographs of the neat PVA film prior to crosslinking (Figure 3a), the crosslinked PVA hydrogel (Figure 3b), and a PVA/PANI-NP composite hydrogel (Figure 3c) are shown for comparison, illustrating clear macroscopic differences among the three systems. The thermal crosslinking process, which is necessary to prevent dissolution of the hydrophilic polymer in aqueous media, involves temperatures of approximately 150 °C and imparts a characteristic yellowish coloration to the neat PVA hydrogel, absent in the uncrosslinked film. This chromatic change is attributed to partial thermal degradation or dehydration of PVA side chains at elevated temperatures, consistent with previously reported observations. In contrast, the PVA/PANI-NP composite hydrogels display the characteristic green coloration of PANI in the emeraldine salt state, reflecting the acidic pH of the incorporated nanoparticle prior to crosslinking [18].

The chemical nature of the crosslinking was confirmed by ATR-FTIR spectroscopy (Figure S3). The reduction of the -OH stretching band (3200–3500 cm^−1^) and the appearance of a new carbonyl band at approximately 1720 cm^−1^, attributed to ester C=O stretching [13], suggesting the formation of covalent ester linkages between the carboxylic groups of citric acid and the hydroxyl groups of PVA upon thermal treatment.

**Figure 4:**
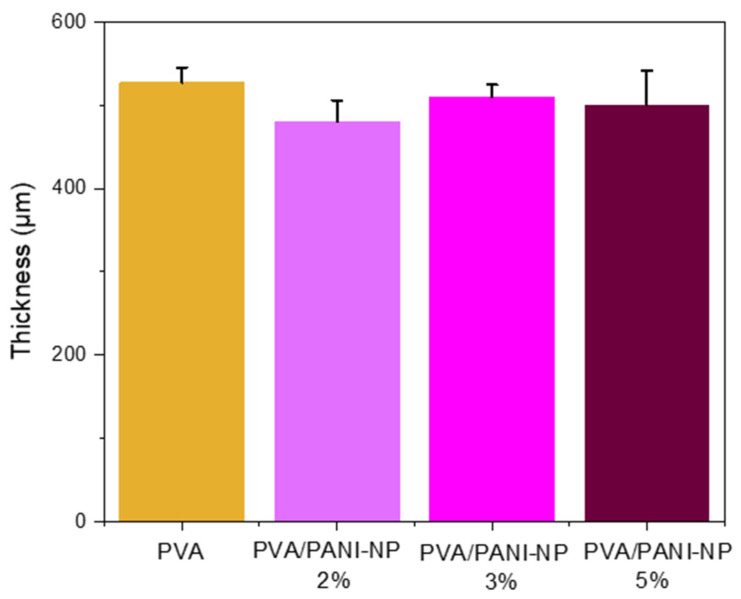
Thickness of neat PVA and PVA/PANI-NP composite hydrogels. Each sample was measured at eight different locations.

The reproducibility of the fabrication method is evidenced by the consistent thickness achieved across samples. Both the neat PVA and composite hydrogels present comparable thicknesses of approximately 500 µm (Figure 4), confirming the PANI-NP incorporation does not significantly alter the film-forming process and that the resulting materials are of adequate thickness for easy handling and further characterization.

**Figure 5:**
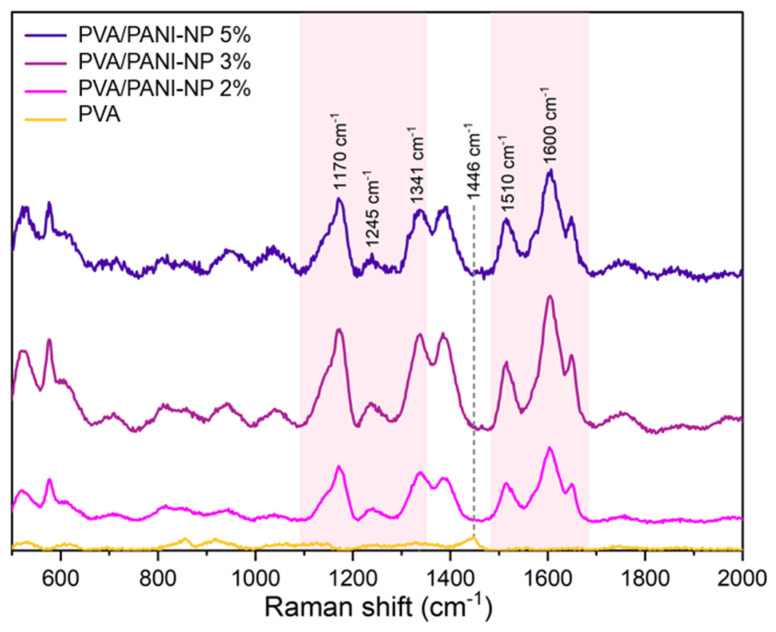
Raman spectra of neat PVA and PVA/PANI-NP composites with increasing PANI nanoparticle content (2, 3, and 5% *w*/*w*).

Raman spectra provided clear evidence for the incorporation of PANI-NP into the PVA matrix (Figure 5). The spectrum of neat PVA showed only a weak, low-intensity band near 1430–1450 cm^−1^ attributable to CH_2_ deformation modes of the PVA backbone. In contrast, the PVA/PANI-NP composite hydrogels exhibited the characteristic vibrational fingerprint of polyaniline in the 1100–1700 cm^−1^ region. Bands observed at approximately 1170, 1245, 1341, 1510, and 1600 cm^−1^, are assigned to aromatic C–H vibrations, C–N stretching of polaronic units, C–N^+^ polaronic vibrations, N–H deformation, and C–C stretching of benzenoid rings, respectively [37,38]. Their intensity increased systematically with PANI-NP loading from 2 to 5% *w*/*w*, confirming composition-dependent incorporation of the nanofiller into the polymer matrix. No new Raman bands were observed, and no clear, systematic peak shift could be unambiguously resolved from the spectra. Accordingly, the Raman data support progressive PANI-NP incorporation but do not provide direct evidence for the formation of new covalent bonds. Instead, the PVA/PANI-NP interaction is more consistent with weak forces, such as hydrogen bonding and dipolar association, rather than the formation of new covalent linkages.

**Figure 6:**
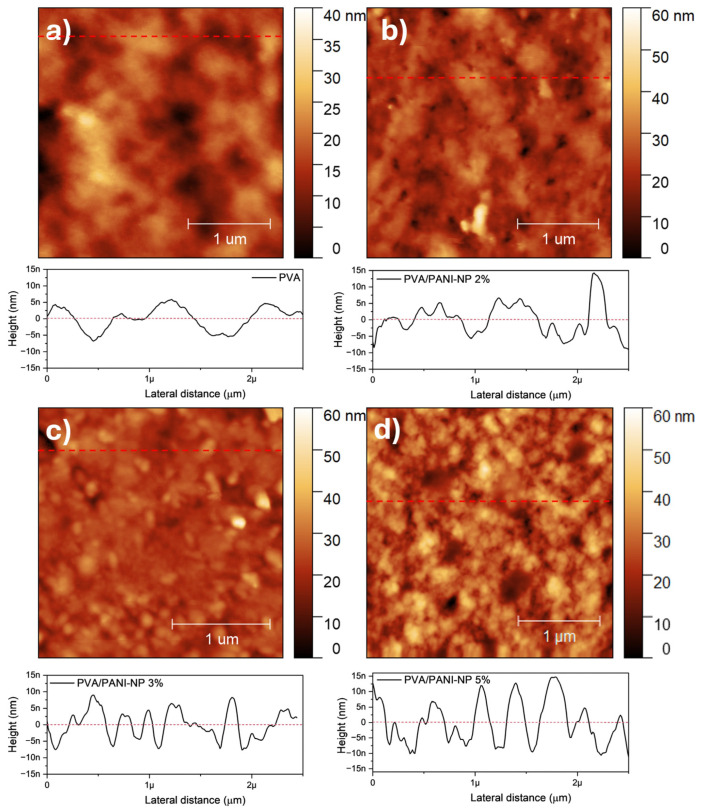
AFM topography maps and representative height profiles of neat PVA and PVA–PANI composite films. Topographic images of (**a**) PVA, (**b**) PVA–PANI 2%, (**c**) PVA–PANI 3%, and (**d**) PVA–PANI 5% films acquired over 2.5 × 2.5 µm^2^ areas.

AFM characterization revealed that incorporation of PANI-NP progressively reorganized the PVA hydrogel surface at the nanoscale. PVA without PANI-NP displayed a relatively smooth and homogeneous topography, whereas the composite films developed increasingly structured and heterogeneous surface textures with increasing nanofiller content as shown in Figure 6. Despite these differences, all samples remained within the nanometric roughness regime, with root-mean-square roughness (Sq) values ranging from 4.0 to 6.9 nm (Figure S4). Specifically, Sq increased from 4.4 nm for PVA to 5.2 nm for PVA/PANI-NP 2%, decreased to 4.0 nm for PVA/PANI-NP 3%, and reached its maximum value at 6.9 nm for PVA/PANI-NP 5%. As Sq reflects the amplitude of vertical surface fluctuations relative to the mean plane, these results indicate that the PVA/PANI-NP 5% composite presented the most topographically heterogeneous surface, while the 3% film remained comparatively smooth despite exhibiting notable mechanical contrast, as discussed below. Surface topography alone is therefore insufficient to account for the mechanical trends observed across the composite series.

**Figure 7:**
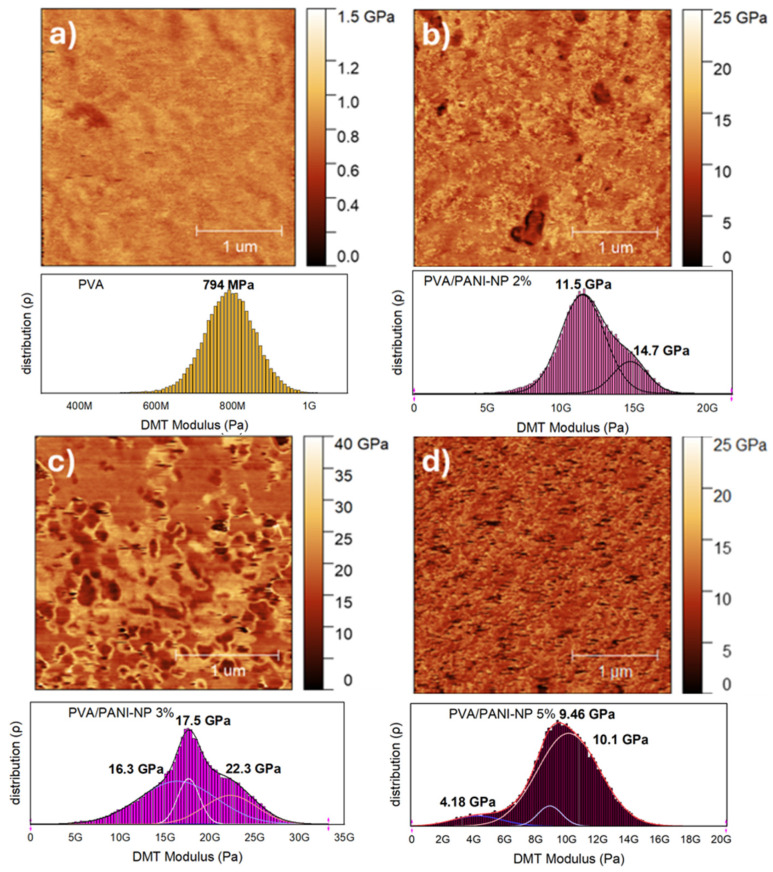
PF-QNM DMT modulus maps and corresponding pixel-value distributions of (**a**) PVA, (**b**) PVA/PANI-NP 2%, (**c**) PVA/PANI-NP 3%, and (**d**) PVA/PANI-NP 5% films, together with the corresponding pixel-value distributions extracted from each map.

PF-QNM modulus maps revealed that the incorporation of PANI-NP exerted a substantially stronger influence on local stiffness than on surface roughness (Figure 7). Neat PVA hydrogel exhibited a narrow, unimodal modulus distribution centered at 794 MPa, consistent with a mechanically uniform matrix. Upon addition of 2% *w*/*w* PANI-NP, the distribution shifted abruptly into the GPa range and became bimodal, with peaks at 11.1 and 14.7 GPa, reflecting the emergence of mechanically distinct nanoscale regions within the polymer matrix. This effect was most pronounced at 3% *w*/*w* PANI-NP, where the modulus distribution became clearly multimodal, with peaks at 16.3, 17.5, and 22.3 GPa, indicative of a highly heterogeneous nanomechanical landscape. At 5% *w*/*w* PANI-NP, the distribution shifted back toward lower values, with maxima at 4.18, 9.46, and 10.1 GPa, demonstrating that the highest nanoparticle loading did not yield the greatest local reinforcement, a result that may reflect nanofiller aggregation or a less efficient stress-transfer interphase at elevated PANI-NP concentrations [38]. This non-monotonic trend is further corroborated by the RMS of the modulus channel (Figure S4a,b), which quantifies the spatial heterogeneity of the nanomechanical response. The modulus RMS increased from approximately 0.1 GPa in neat PVA to 2.2 GPa in PVA/PANI-NP 2%, reached a maximum of 4.4 GPa in PVA/PANI-NP 3%, and decreased to 2.4 GPa in PVA/PANI-NP 5%. The formulation exhibiting the highest characteristic modulus values thus also displayed the greatest mechanical heterogeneity, confirming that the PVA/PANI-NP 3% hydrogel composite contains the strongest contrast between soft and stiff nanoscale regions. Taken together, the elevated local modulus and high modulus RMS observed at 3% *w*/*w* PANI-NP loading suggest the formation of a nanoscale interphase architecture that facilitates more efficient load transfer than that achieved at higher PANI-NP concentration, consistent with an optimal nanofiller dispersion regime.

The PeakForce error maps (Figure S5) are consistent with this interpretation. Although included here primarily as supporting data, they reveal rapid local transitions in tip–sample interaction and become progressively more structured in the PANI-NP containing hydrogels, particularly at 3% and 5% loading. This confirms that nanoparticle addition increases the nanoscale surface complexity, both morphologically and mechanically. The adhesion response evolved in the opposite direction. The most probable adhesion force decreased monotonically with increasing PANI content, from approximately 80 nN for neat PVA to ~40 nN for PVA/PANI-NP 2%, ~33 nN for PVA/PANI-NP 3%, and ~15–17 nN for PVA/PANI-NP 5%. The RMS of the adhesion channel (Figure S4c) followed the same trend, decreasing from ~10.3 nN to 9.2, 6.8, and 3.7 nN, respectively. Together, these results indicate that PANI-NP progressively reduces both the magnitude and spatial variability of tip–sample adhesion. Because adhesion in PF-QNM is governed by local surface chemistry, surface energy, and contact area, the observed reduction suggests that increasing nanoparticle loading alters the outermost interfacial character of the composites, rendering them less adhesive than the neat PVA hydrogel.

**Figure 8:**
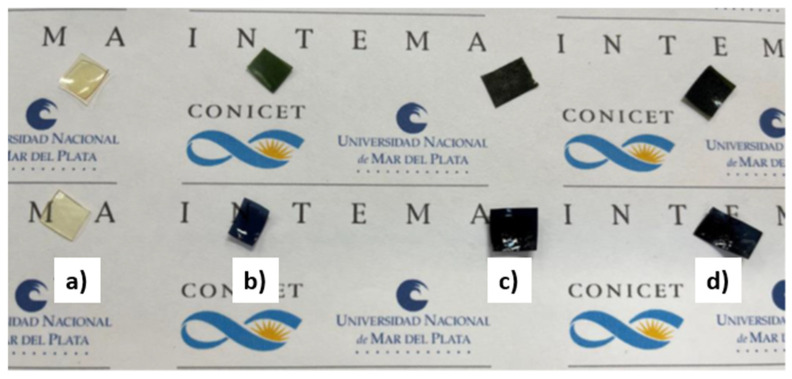
Photographs showing the pH-dependent response of different systems under acidic and basic conditions. (**a**) PVA hydrogel; (**b**) PVA/PANI-NP composite hydrogel (2%); (**c**) PVA/PANI-NP composite hydrogel (3%) and (**d**) PVA/PANI-NP composite hydrogel (5%).

Given the electroactive properties of PANI-NP, particularly their pH-dependent redox switching, analogous behavior is expected in the developed composites [28]. Beyond the Raman spectroscopy results confirming the nanoparticle incorporation into the PVA network, a simple visual test was performed to verify whether PANI-NP retain their electroactive behavior within the composite matrix. Upon immersion in 0.1 mol L^−1^ NaOH, the PANI-ES converts to base emeraldine, as shown in Figure 8. This transition is fully reversible: subsequent immersion in HCl solution (Video S1) restores the characteristic green coloration of the emeraldine salt state. Notably, no particle leaching into the surrounding medium was observed throughout these experiments, supporting the existence of interfacial interactions between the nanoparticles and the polymer matrix. Taken together, these findings confirm the electroactive behavior of PANI is preserved in nanoparticulate form within the composite hydrogels.

Table 2 presents the equilibrium swelling values measured after 12 h for each system. Neat PVA hydrogel exhibited a maximum swelling of 247 ± 10%, whereas incorporation of PANI-NP significantly reduced this value to approximately 140% at loading of 2% and 3%. This reduction can be attributed to the interactions between PANI-NP and the PVA chains, which restrict chain mobility and decrease the free volume available for water uptake within the network. The PVP shell surrounding the nanoparticles, retained after synthesis and purification, may further contribute to these interfacial interactions through hydrogen bonding with the hydroxyl groups of PVA, thereby reinforcing the network structure. However, at 5% PANI-NP loading, swelling increased relative to the 2% and 3% composites, although it remained below the value recorded for neat PVA. This non-monotonic behavior is likely attributable to the less homogeneous nanoparticle organization at higher concentrations, leading to the formation of agglomerated domains, as suggested by the AFM analysis. Such aggregation would locally disrupt the network architecture and create regions of greater free volume, facilitating water uptake. Collectively, these results indicate that swelling behavior in these composites is governed primarily by bulk network structure rather than surface properties alone.

**Figure 9:**
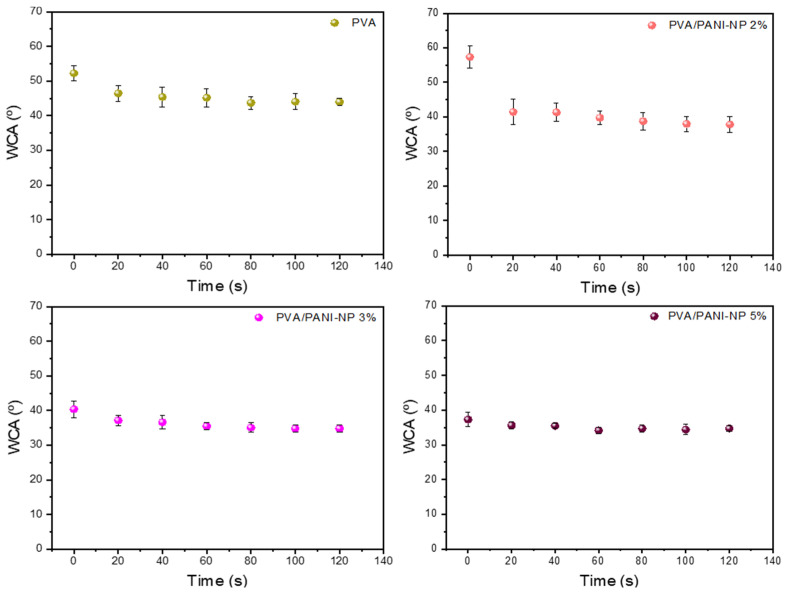
Water contact angle (WCA) of neat PVA and PVA/PANI NP composite hydrogels as a function of time (n = 5).

Surface hydrophilicity was evaluated by WCA measurements as a function of time as shown in Figure 9. Although initial values (t = 0 s) showed some variability attributable to drop deposition dynamics, equilibrium was reached at approximately 120 s and these values were used for comparison. Neat PVA hydrogel presented a WCA of ~44°, confirming its hydrophilic character. Incorporation of PANI-NP produced a slight but consistent decrease in WCA across all composite formulations, with equilibrium values in the range of 35–38°, indicating enhanced surface hydrophilicity. This trend is attributed to the presence of functional groups in PANI that increase water affinity at the composite surface. In particular, the PVA/PANI-NP 3% and 5% composites displayed similar WCA values, suggesting a saturation effect at higher nanoparticle contents. The PVA/PANI-NP 2% composite presented an intermediate value, lower than neat PVA but marginally higher than the other composites. These surface findings appear, at first glance, to conflict with the swelling results, which showed that the 2% and 3% composites absorbed significantly less water than neat PVA despite their greater surface hydrophilicity. This apparent contradiction underscores that bulk water uptake is governed primarily by the internal polymer network architecture, crosslinking density, chain mobility and free volume, rather than by surface affinity alone, a distinction critical to the correct interpretation of hydrogel behavior. The WCA results are further consistent with the AFM analysis, which revealed a progressive reduction in tip-sample adhesion forces with increasing PANI-NP content alongside modifications in surface roughness, collectively reflecting the changes in surface physicochemical properties induced by nanoparticle incorporation.

**Figure 10:**
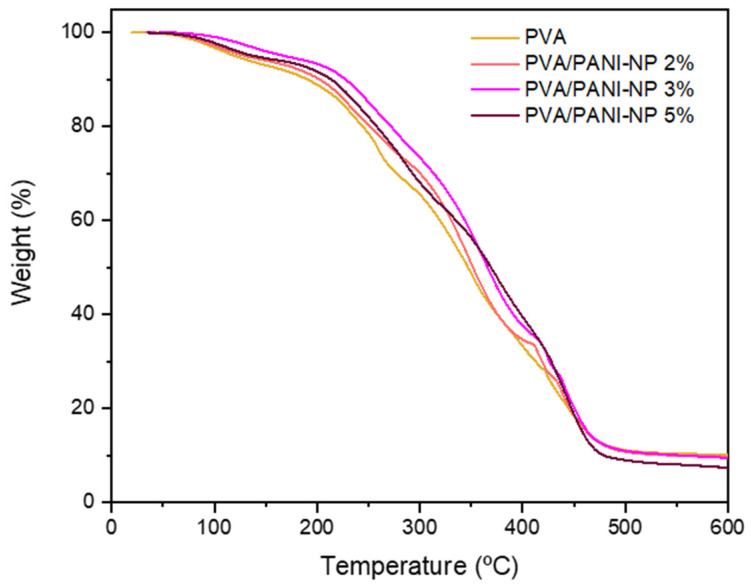
Thermogravimetric analysis (TGA) curves of neat PVA and PVA/PANI-NP composite hydrogels.

The thermal stability of the PVA/PANI-NP composite hydrogels and neat PVA hydrogel was assessed by TGA (Figure 10). All systems displayed broadly similar degradation profile as function of temperature, suggesting that PANI-NP incorporation does not significantly alter the degradation mechanism of the PVA matrix, in agreement with previous reports for this polymer. The principal weight loss occurred in the 250–380 °C range, corresponding to degradation of the PVA chains [11]. Although the typical degradation temperature of PVA is commonly reported at approximately 310–320 °C, a slight shift toward higher temperatures (~320–330 °C) was observed for PVA/PANI-NP 2% and 3% composites, indicative of improved thermal stability [18]. This enhancement may also reflect the contribution of citric acid crosslinking to network cohesion. In contrast, the PVA/PANI-NP 5% composite did not exhibit a comparable shift, likely because the higher nanoparticle content reduces the efficiency of interfacial interactions within the network, consistent with the aggregation behavior inferred from AFM analysis. The residual mass at 600 °C remains within the 8–10% range for all formulations, indicating that the presence of electroactive particles does not significantly affect the char yield. The thermal results align well with the trends observed in the swelling experiments. The PVA/PANI-NP 2% and 3% composites, which exhibited both reduced water uptake and improved thermal stability, appear to benefit from efficient polymer–nanoparticle interfacial interactions that reinforce the network structure. The PVA/PANI-NP 5% composite, by contrast, shows neither enhanced thermal stability nor reduced swelling relative to these lower-loaded systems, further supporting the conclusion that excessive nanoparticle content disrupts network homogeneity and diminishes the beneficial effects of interfacial coupling.

**Figure 11:**
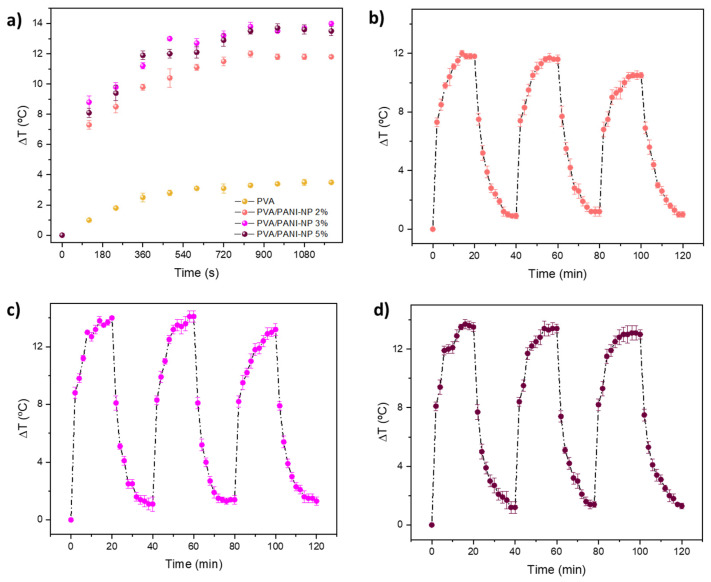
(**a**) Photothermal performance of neat PVA and PVA/PANI-NP composite hydrogels under low-power near-infrared LED irradiation: temperature increase as a function of irradiation time for all systems. (**b**–**d**) Photothermal heating–cooling cycles of the composite hydrogels at different nanoparticle loadings, demonstrating the stability and reproducibility of the response: PVA/PANI-NP 2% (**b**), PVA-PANI-NP 3% (**c**), and PVA/PANI-NP 5% (**d**).

The photothermal response of the nanocomposites and PVA hydrogel under low-power NIR LED irradiation at 850 nm is presented in Figure 11a. Upon irradiation, all three PANI-NP containing composites exhibited a progressive temperature increase that reached a plateau within approximately 15 min. Neat PVA, by contrast, showed a negligible temperature rise of ~3 °C, confirming that the photothermal behavior originates exclusively from the electroactive PANI-NP and not from the polymeric matrix itself. The maximum temperature increases recorded above the initial value were 11.5 °C for PVA/PANI-NP 2% and 13.7 °C for both PVA/PANI-NP 3% and 5%. Although all systems achieved substantial photothermal heating, the temperature profiles of the 3% and 5% systems overlapped closely throughout the irradiation period, with final values that were practically indistinguishable. This convergence suggests that beyond a certain nanoparticle loading, incremental photothermal gains become negligible, a finding consistent with the nanomechanical and thermal analyses, which similarly showed that the PVA/PANI-NP 5% composite did not outperform the PVA/PANI-NP 3% formulation in terms of interfacial efficiency or network organization. The stability and reproducibility of the photothermal response were assessed over three consecutive heating–cooling cycles for each composite formulation (Figure 11b–d). All systems demonstrated a highly reproducible response with no significant loss in photothermal performance across cycles, indicating that PANI-NP retain their light-to-heat conversion activity within the PVA matrix without degradation under the applied irradiation conditions. Collectively, these results demonstrate the potential of PVA/PANI-NP composite hydrogels as stable and efficient photothermal platforms for light-responsive applications.

## 4. Conclusions

Photothermally responsive PVA/PANI-NP composite hydrogels were successfully developed through a straightforward, fully aqueous route using citric acid as a non-toxic crosslinking agent, ensuring safe processing conditions and potential scalability. The successful incorporation of the electroactive PANI-NP into the PVA matrix was confirmed by Raman spectroscopy, and their electroactive properties were preserved within the final composites, as evidenced by their reversible pH-dependent color switching. The resulting hydrogels exhibited homogeneous thickness, thermal stability, tunable swelling behavior, while retaining the inherent hydrophilicity of PVA with a modest increase in surface roughness. Nanomechanical analysis by AFM revealed that PANI-NP incorporation progressively modified local stiffness, surface adhesion and interfacial architecture in a loading-dependent manner, with the most efficient polymer–nanoparticle interactions observed at intermediate loadings. Under low-power NIR LED irradiation at 850 nm, all composite formulations demonstrated a clear photothermal response, with temperature increases of up to ~13.5 °C above the initial value, negligible heating of the neat PVA matrix, and stable performance over three consecutive heating–cooling cycles. Among all formulations evaluated, PVA/PANI-NP 3% offered the most favorable balance between structural integrity, interfacial efficiency, and photothermal performance, making it the most promising candidate for integration in light-responsive systems and smart material platforms.

## Figures and Tables

**Table 1:** Composition of neat PVA and PVA/PANI NP composite hydrogels, including the amounts of each component used in the formulations.

Sample	PVA (%)	Citric Acid (%)	Glycerol (%)	PANI-NP (g)
PVA hydrogel	10	20	25	-
PVA/PANI-NP 2%	10	20	25	0.0288
PVA/PANI-NP 3%	10	20	25	0.0432
PVA/PANI-NP 5%	10	20	25	0.0720

**Table 2:** Maximum swelling (%) of neat PVA and PVA/PANI NP composite hydrogels (mean ± standard deviation, n = 5).

Sample	Equilibrium Swelling (%)
PVA hydrogel	247 ± 10
PVA/PANI-NP 2%	142 ± 11
PVA/PANI-NP 3%	141 ± 8
PVA/PANI-NP 5%	206 ± 20

## Data Availability

The original contributions presented in this study are included in the article/Supplementary Material. Further inquiries can be directed to the corresponding author.

## Supplementary Materials

The following supporting information can be downloaded at https://www.mdpi.com/article/10.3390/polym18131638/s1, Figure S1. ATR-FTIR spectrum of PANI-NP showing the characteristic absorption bands of polyaniline in the ES form. Figure S2. TGA curve of PANI-NP under nitrogen atmosphere. Figure S3. ATR-FTIR spectra of PVA hydrogel before and after thermal treatment, showing the changes in the characteristic absorption bands upon chemical crosslinking with citric acid. Figure S4. Root mean square (RMS) values extracted from the (a) height, (b) DMT modulus, and (c) adhesion channels for neat PVA and PVA/PANI-NP composites. For the height channel, the RMS corresponds to the surface roughness (Sq). For the nanomechanical channels, the RMS values serve as descriptors of spatial heterogeneity across the scanned area, rather than as direct measures of the respective property magnitude. Figure S5. (a) Adhesion and (b) Peak Force error images of PVA, PVA/PANI-NP 2%, PVA/PANI-NP 3%, and PVA/PANI-NP 5% films acquired over 2.5 × 2.5 µm^2^ areas. Figure S6. Pixel-value distributions of adhesion extracted from the PF-QNM maps. Video S1. Electronic state transition of PVA/PANI-NP materials given by the conductive polymer incorporation.
