# Supplementary material for: Photothermally Responsive Poly(vinyl alcohol)/Polyaniline Nanoparticle Composite Hydrogels Prepared by a Facile Aqueous Route
Source: Polymers (Basel). 2026 Jul 1;18(13):1638. doi: 10.3390/polym18131638 (PMC13364188; doi:10.3390/polym18131638)
Supplement: Supplementary file 1 [file polymers-18-01638-s001.zip › S.I. Battaglia et al. POLYMERS revision.pdf]

SUPPORTING INFORMATION

Photothermally Responsive Poly(vinyl alcohol)/Polyaniline Nanoparticle Composite Hydrogels Prepared by a Facile Aqueous Route

Ernesto S. Battaglia [1,†], Eduart Gutiérrez-Pineda [2,†], César A. Barbero [3], Gustavo A. Abraham [1,4], Sergio E. Moya [2,*] and Silvestre Bongiovanni Abel [1,4,*]

[1] Research Institute for Materials Science and Technology (INTEMA), National University of Mar del Plata (UNMdP)-National Scientific and Technical Research Council (CONICET), Av. Colón 10850, Mar del Plata 7600, Argentina; battaglia@fi.mdp.edu.ar (E.S.B.); gabraham@fi.mdp.edu.ar (G.A.A.)

[2] Center for Cooperative Research in Biomaterials (CIC biomaGUNE), Basque Research and Technology Alliance (BRTA), Paseo Miramon 182, 20014 Donostia San Sebastián, Spain; egutierrez@cicbiomagune.es

[3] Research Institute for Energy Technologies and Advanced Materials (IITEMA), National University of Rio Cuarto (UNRC)-National Scientific and Technical Research Council (CONICET), Ruta Nacional N° 36 Km 601, Río Cuarto 5800, Argentina; cbarbero@exa.unrc.edu.ar

[4] Department of Chemical and Food Engineering, Faculty of Engineering, National University of Mar del Plata (UNMdP), Av. Juan B. Justo 4302, Mar del Plata 7600, Argentina

* Correspondence: smoya@cicbiomagune.es (S.E.M.); bongiovanniabel.s@fi.mdp.edu.ar (S.B.A.)

† These authors contributed equally to this work.

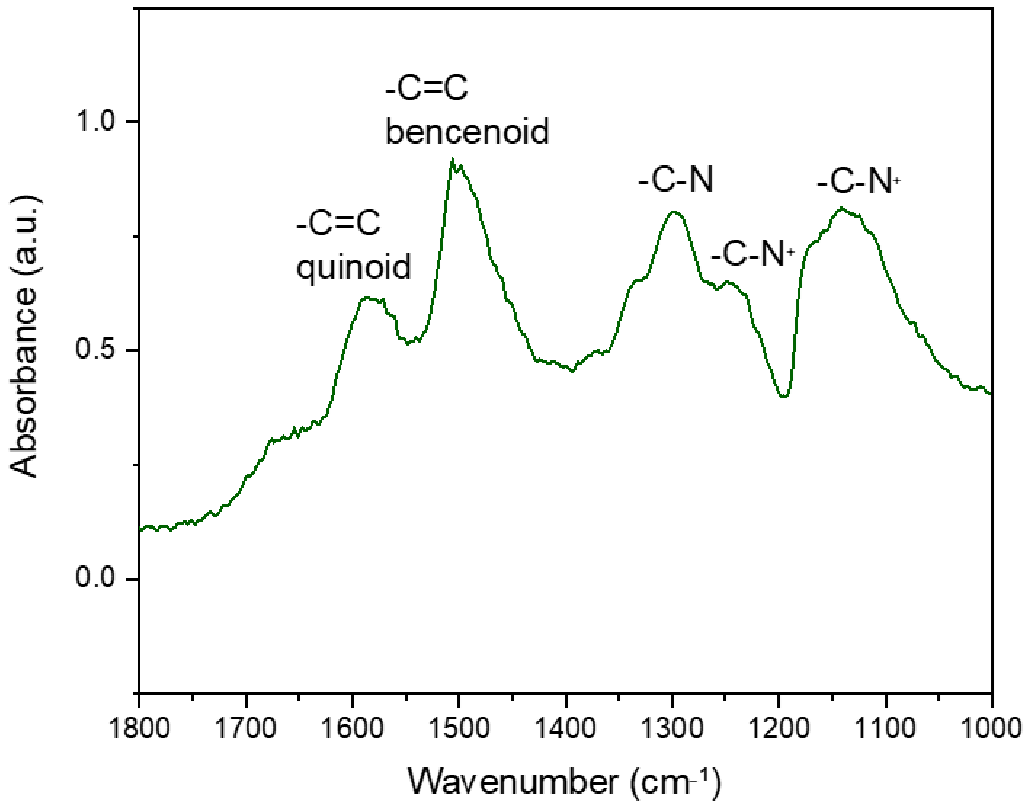

Figure S1. ATR-FTIR spectrum of PANI-NP showing the characteristic absorption bands of polyaniline in the ES form.

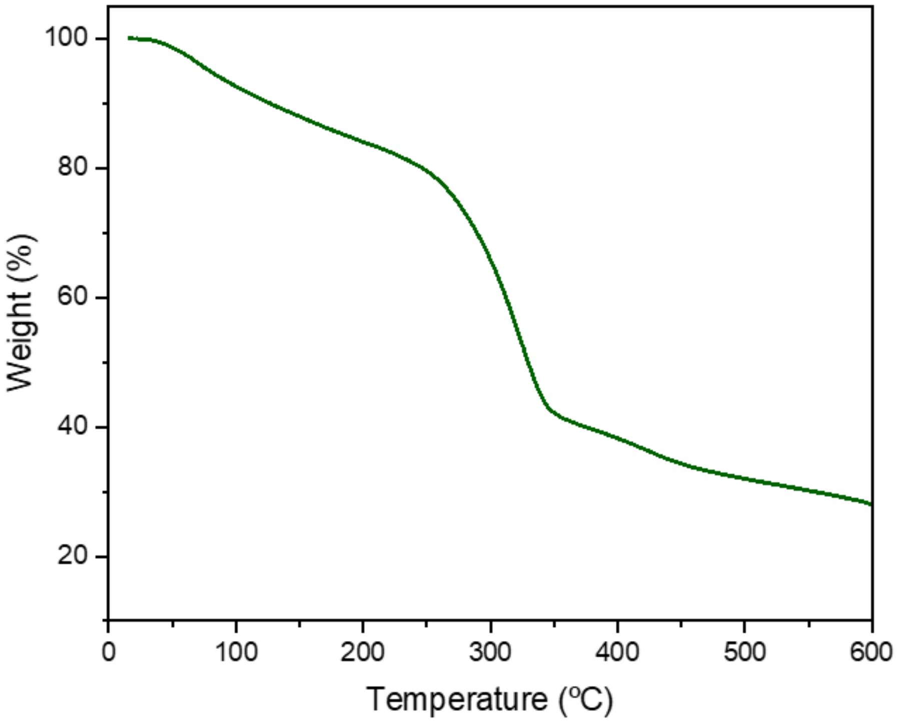

Figure S2. TGA curve of PANI-NP under nitrogen atmosphere.

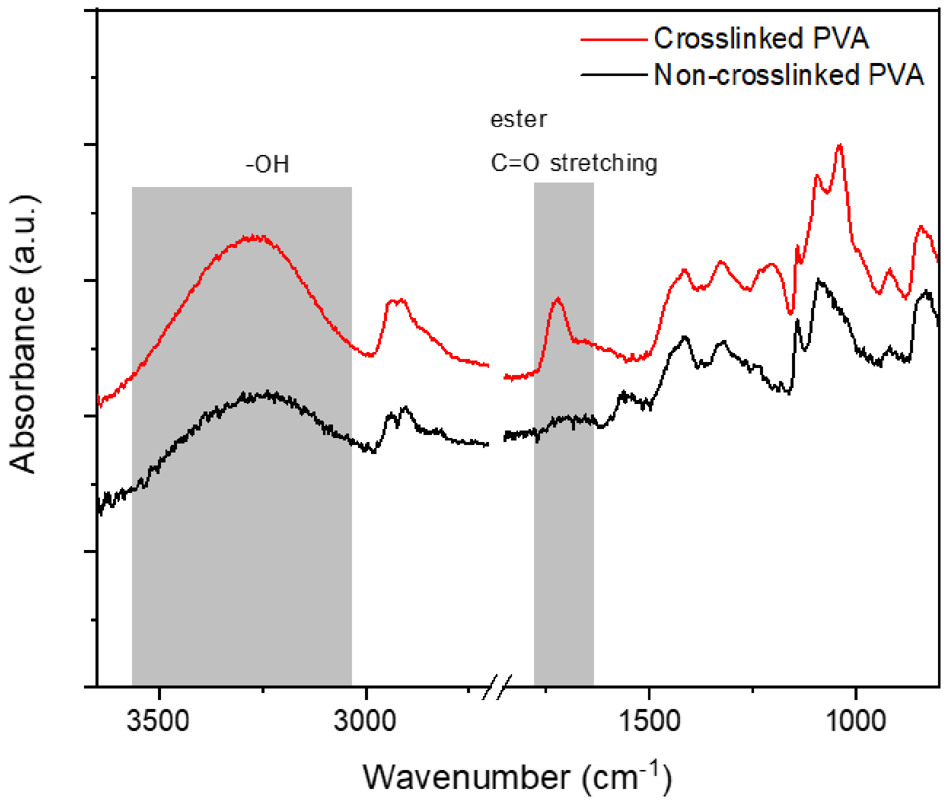

Figure S3. ATR-FTIR spectra of PVA hydrogel before and after thermal treatment, showing the changes in the characteristic absorption bands upon chemical crosslinking with citric acid.

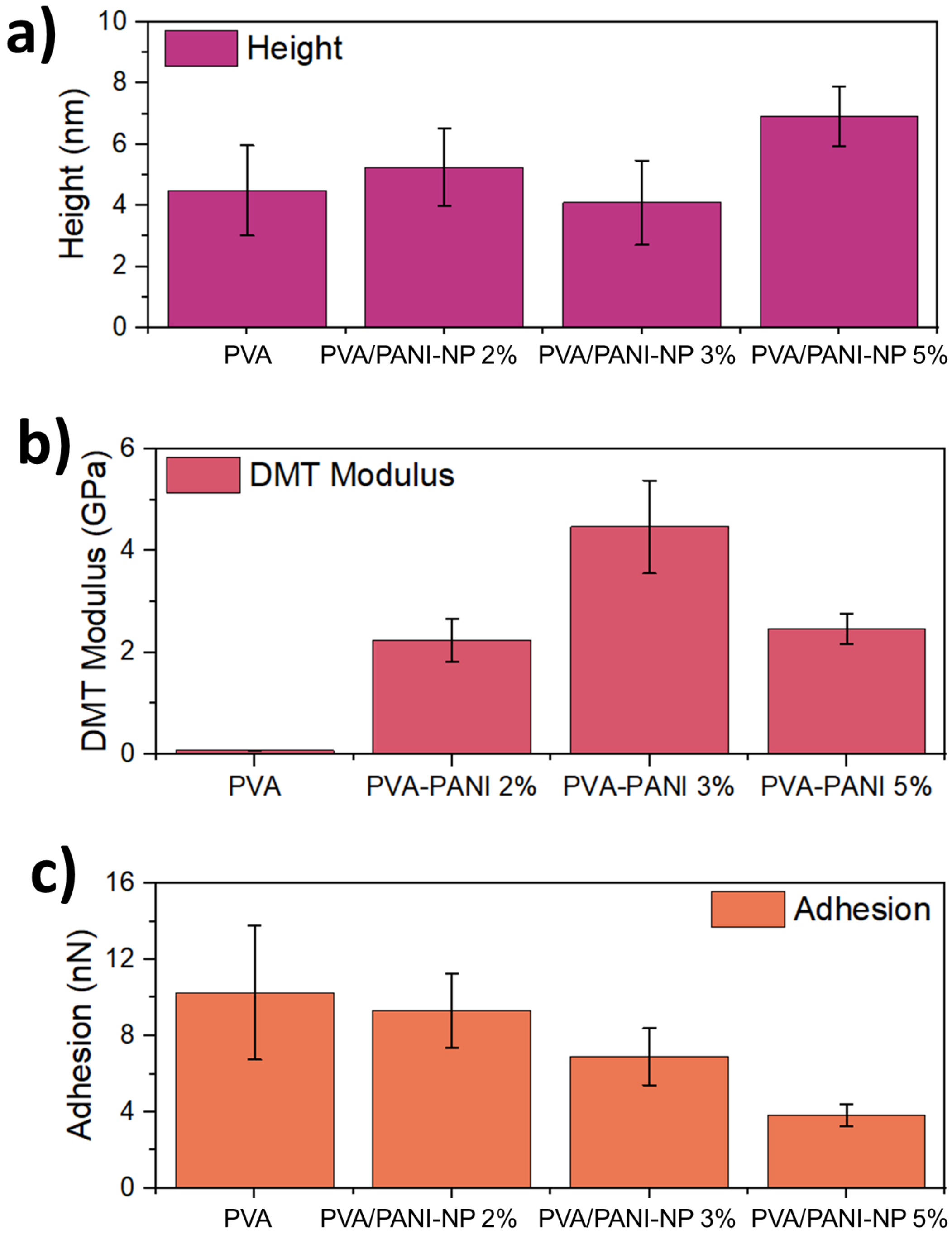

Figure S4. Root mean square (RMS) values extracted from the a) height, b) DMT modulus, and c) adhesion channels for neat PVA and PVA/PANI-NP composites. For the height channel, the RMS corresponds to the surface roughness (Sq). For the nanomechanical channels, the RMS values serve as descriptors of spatial heterogeneity across the scanned area, rather than as direct measures of the respective property magnitude.

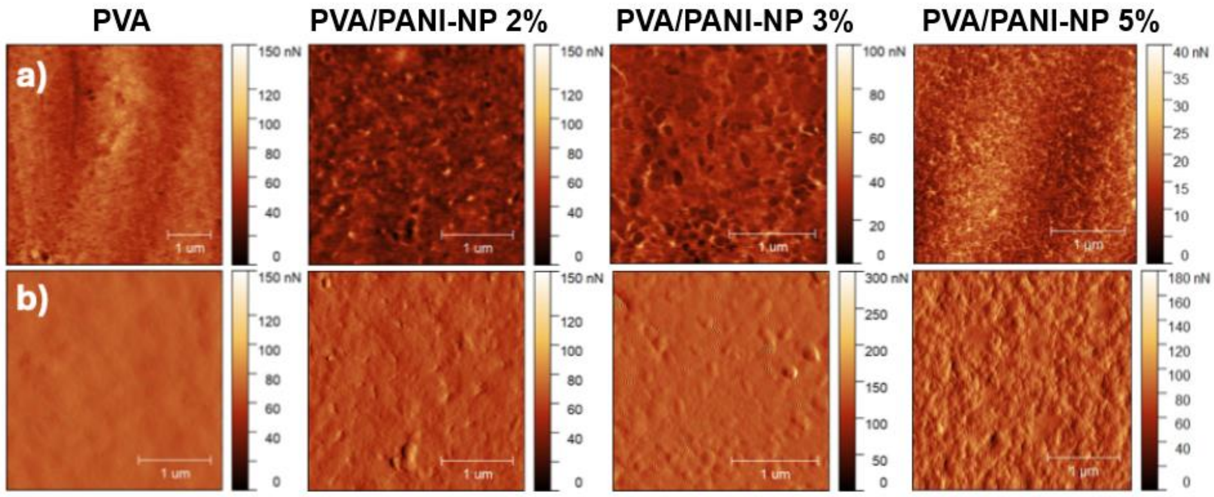

Figure S5. a) Adhesion and b) Peak Force error images of PVA, PVA/PANI-NP 2%, PVA/PANI-NP 3%, and PVA/PANI-NP 5% films acquired over 2.5 × 2.5 µm² areas.

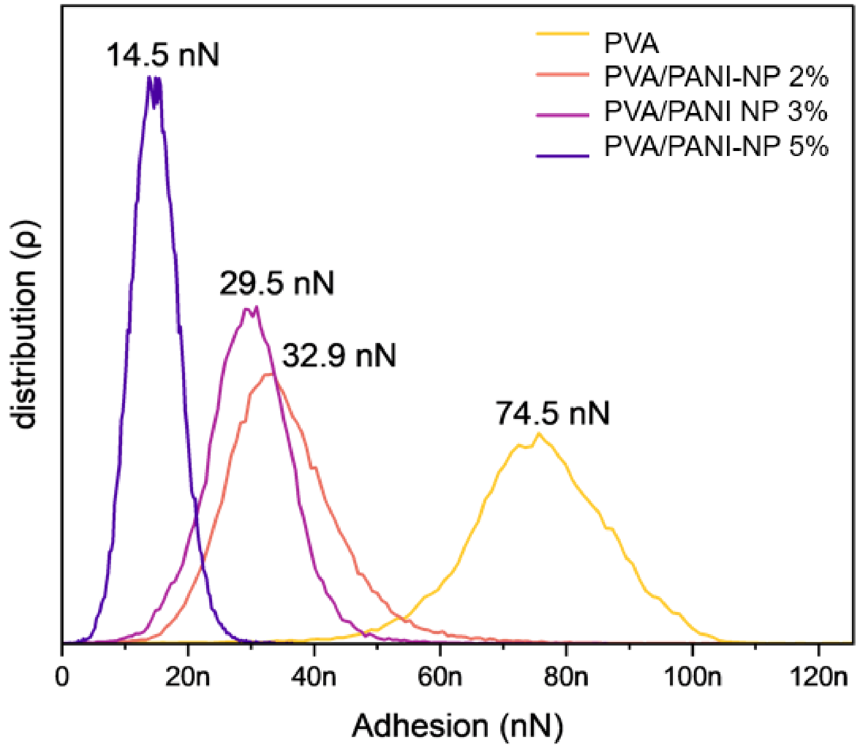

Figure S6. Pixel-value distributions of adhesion extracted from the PF-QNM maps
