# Supplementary material for: Photothermally Responsive Poly(vinyl alcohol)/Polyaniline Nanoparticle Composite Hydrogels Prepared by a Facile Aqueous Route
Source: Polymers (Basel). 2026 Jul 1;18(13):1638. doi: 10.3390/polym18131638 (PMC13364188; doi:10.3390/polym18131638)
Supplement: Supplementary file 1 [file polymers-18-01638-s001.zip › VIDEO S1.pptx]

## Slide 1
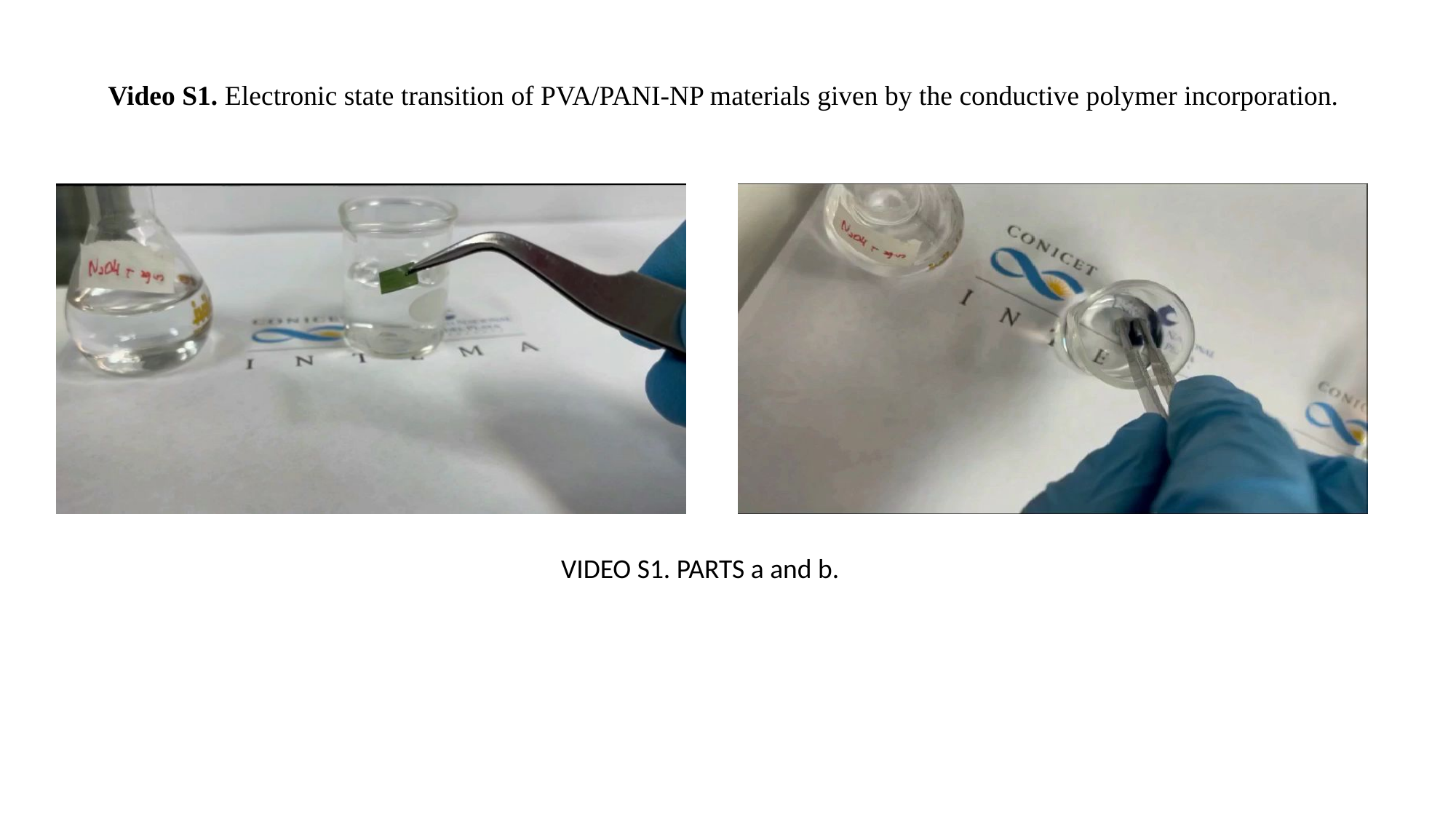

Video S1. Electronic state transition of PVA/PANI-NP materials given by the conductive polymer incorporation.
VIDEO S1. PARTS a and b.
