# Supplementary figures and images for: Photothermally Responsive Poly(vinyl alcohol)/Polyaniline Nanoparticle Composite Hydrogels Prepared by a Facile Aqueous Route
Source: Polymers (Basel). 2026 Jul 1;18(13):1638. doi: 10.3390/polym18131638 (PMC13364188; doi:10.3390/polym18131638)

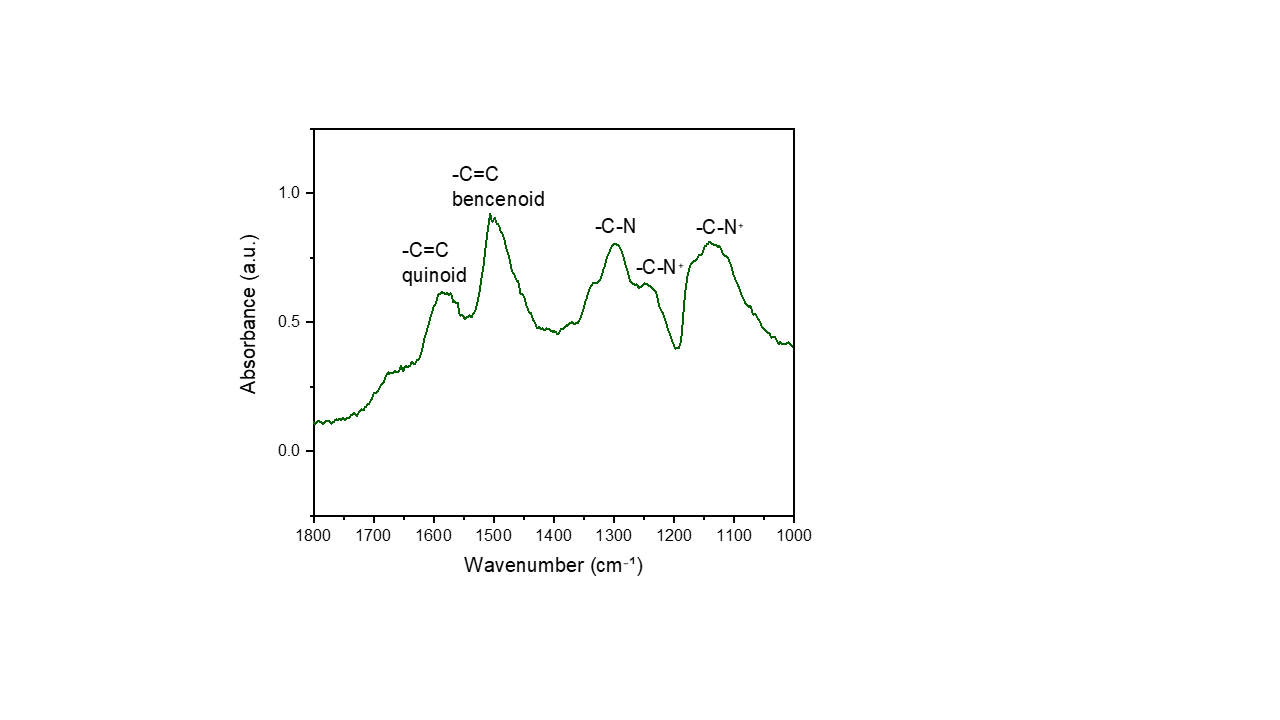

Supplement: Supplementary file 1 [file polymers-18-01638-s001.zip › Figure S1.tif]

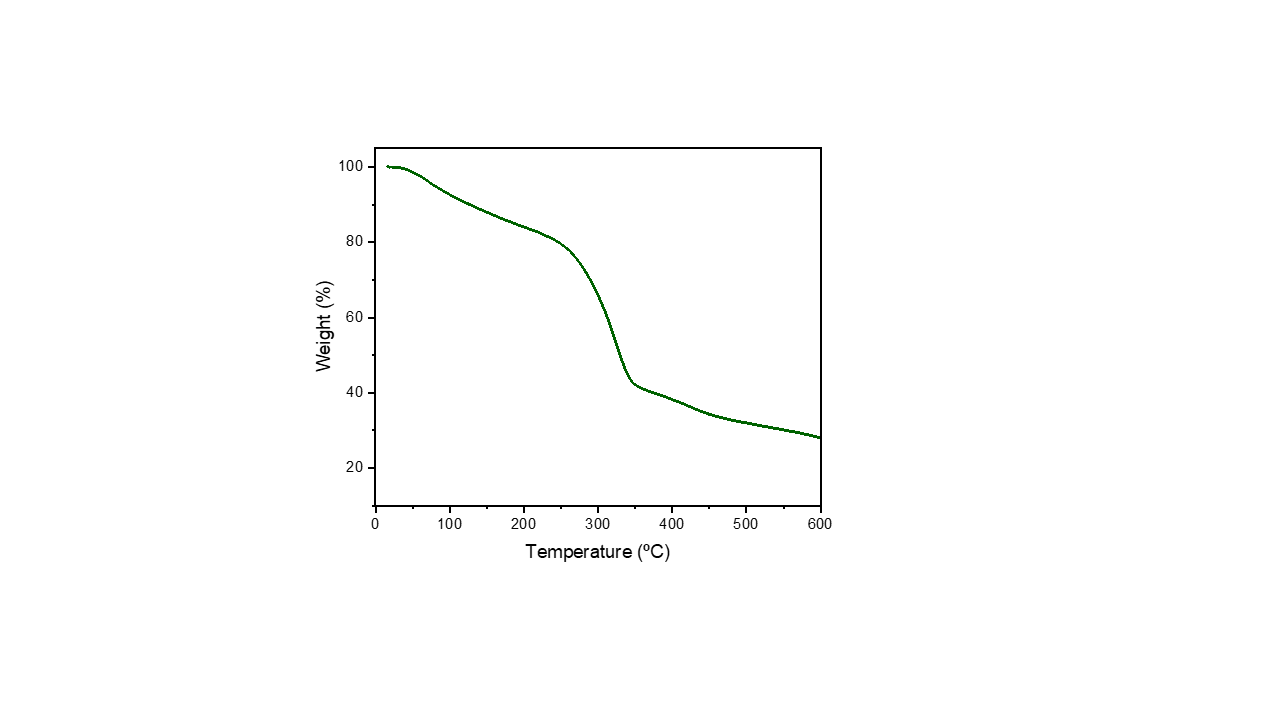

Supplement: Supplementary file 1 [file polymers-18-01638-s001.zip › Figure S2.tif]

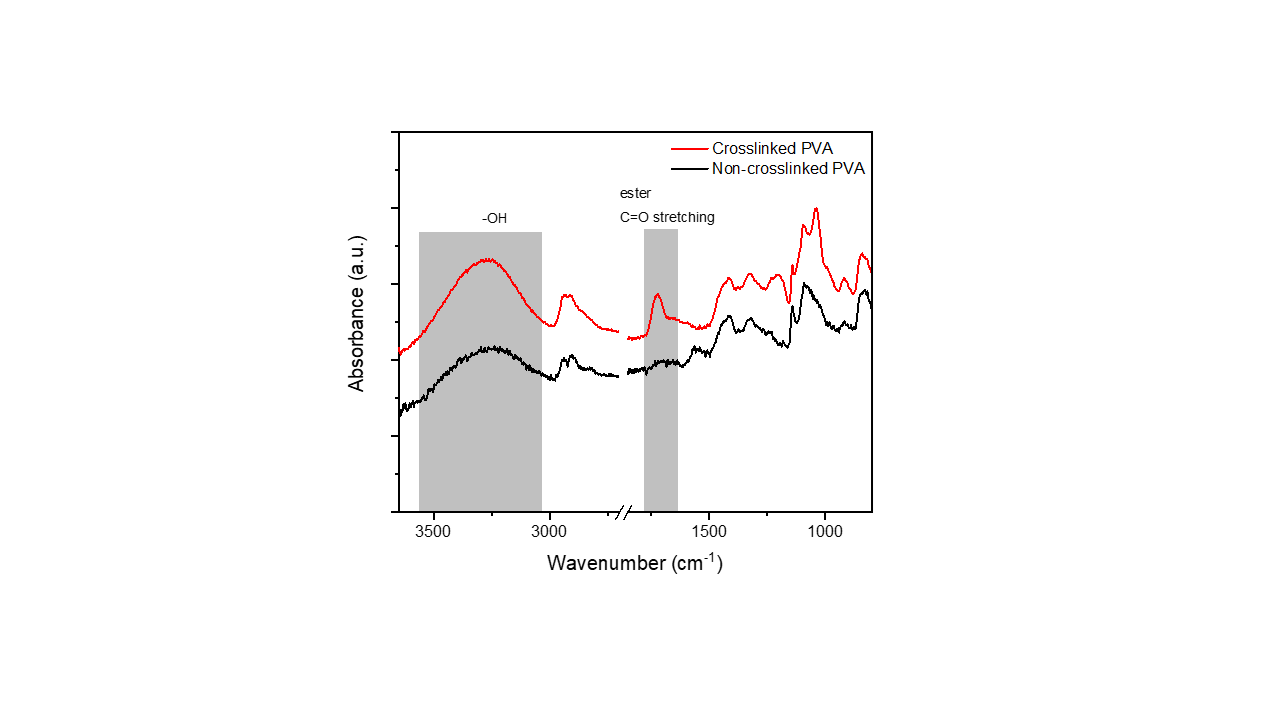

Supplement: Supplementary file 1 [file polymers-18-01638-s001.zip › Figure S3.tif]

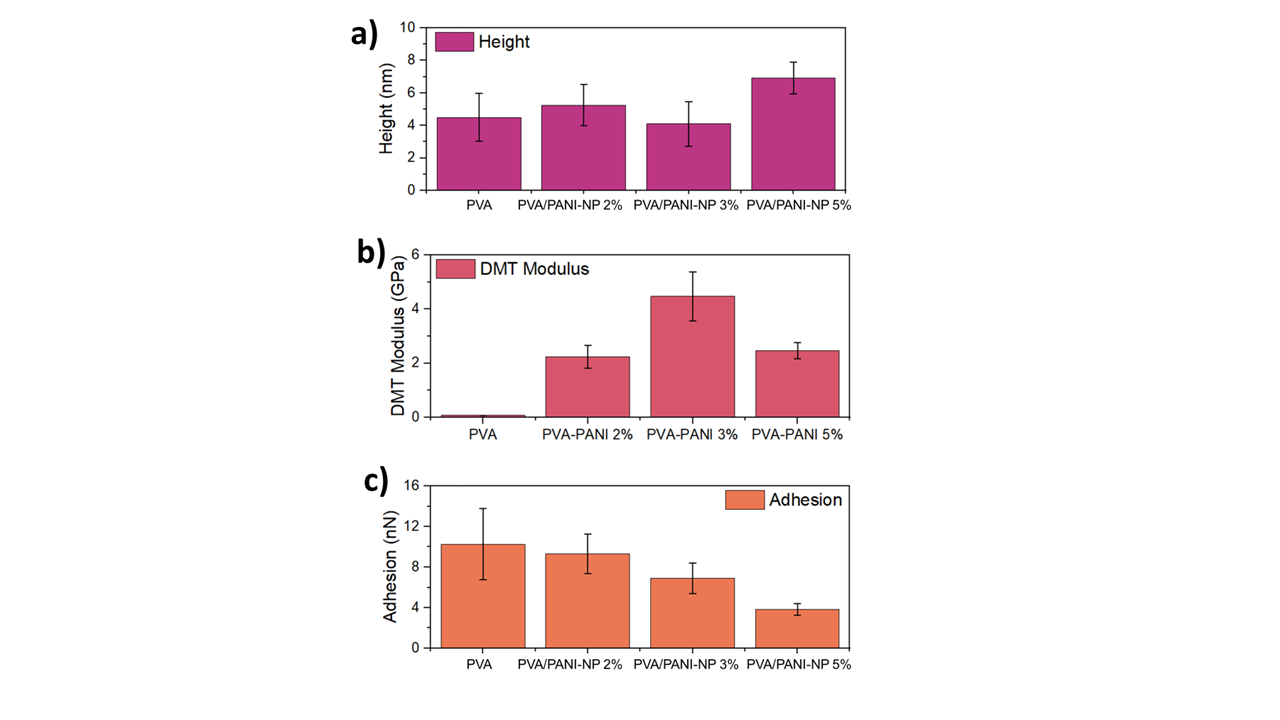

Supplement: Supplementary file 1 [file polymers-18-01638-s001.zip › Figure S4.TIF]

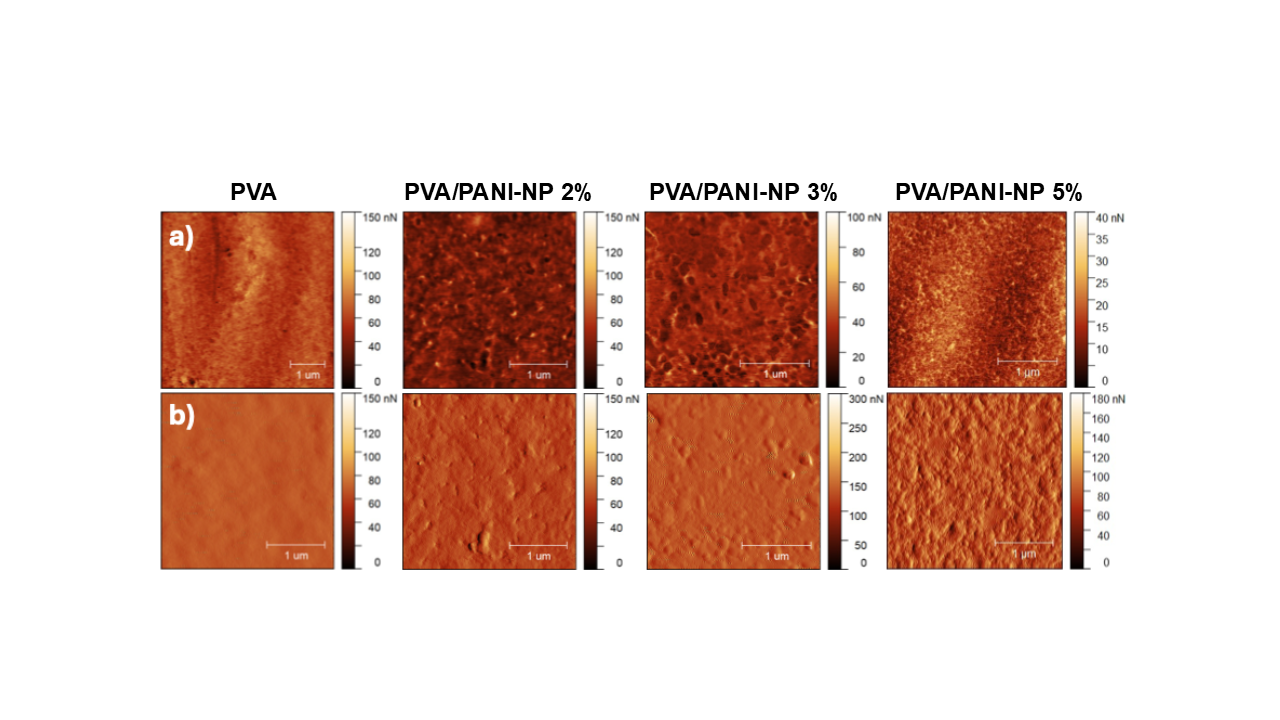

Supplement: Supplementary file 1 [file polymers-18-01638-s001.zip › Figure S5.TIF]

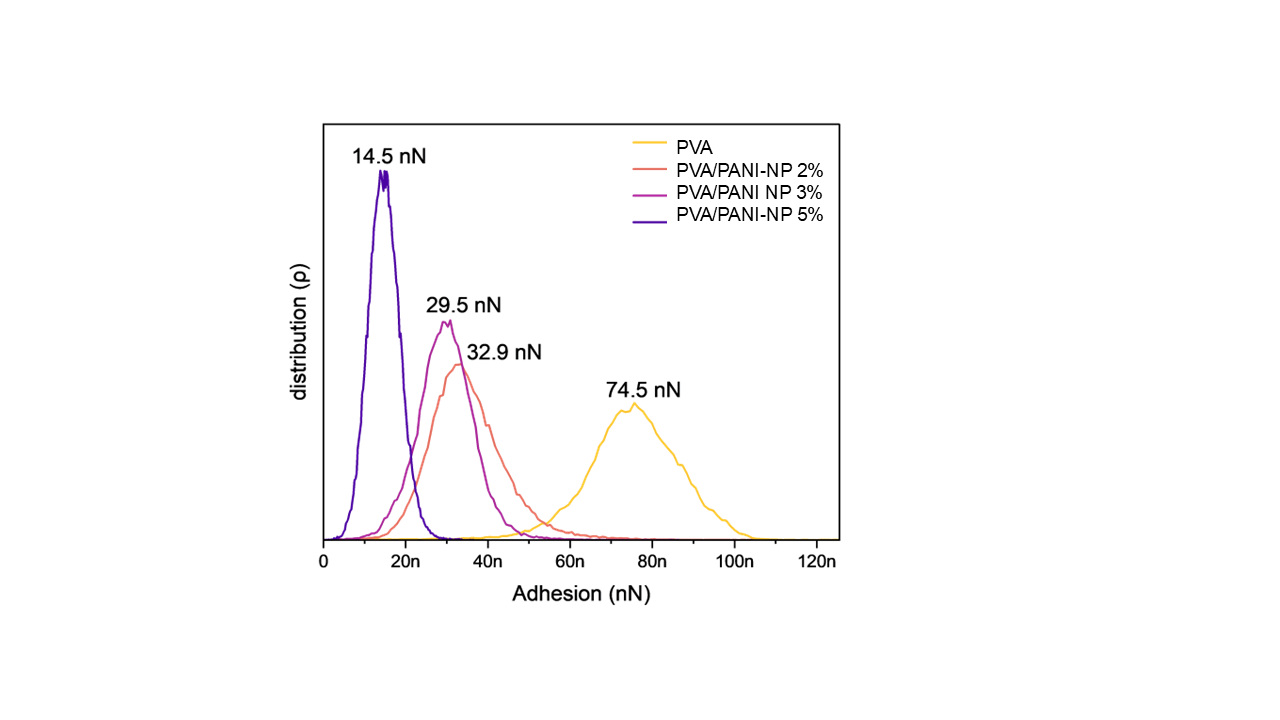

Supplement: Supplementary file 1 [file polymers-18-01638-s001.zip › Figure S6.TIF]
